# A new species of earth snake (Dipsadidae, *Geophis*) from Mexico

**DOI:** 10.3897/zookeys.610.8605

**Published:** 2016-08-11

**Authors:** Luis Canseco-Márquez, Carlos J. Pavón-Vázquez, Marco Antonio López-Luna, Adrián Nieto-Montes de Oca

**Affiliations:** 1 Museo de Zoología and Departamento de Biología Evolutiva, Facultad de Ciencias, Universidad Nacional Autónoma de México, Cd. Universitaria, Del. Coyoacán, Mexico 04510, Ciudad de México, Mexico; 2 División Académica de Ciencias Biológicas, Universidad Juárez Autónoma de Tabasco, km 0.5 carretera Villahermosa-Cárdenas, entronque con Bosques de Saloya, Villahermosa, Tabasco, Mexico

**Keywords:** Dipsadidae, Geophis dubius group, Geophis duellmani, Geophis turbidus, Mexico, New species, Puebla, Veracruz

## Abstract

A new species of the *Geophis dubius* group is described from the mountains of the Sierra Zongolica in west-central Veracruz and the Sierra de Quimixtlán in central-east Puebla. The new species is most similar to *Geophis duellmani* and *Geophis turbidus*, which are endemic to the mountains of northern Oaxaca and the Sierra Madre Oriental of Puebla and Hidalgo, respectively. However, the new species differs from *Geophis duellmani* by the presence of postocular and supraocular scales and from *Geophis turbidus* by having a bicolor dorsum. With the description of the new species, the species number in the genus increases to 50 and to 12 in the *Geophis dubius* group. Additionally, a key to the species of the *Geophis dubius* group is provided.

## Introduction

With 49 recognized species (Pavón-Vázquez et al. 2013), the colubrid genus *Geophis* (Dipsadidae) is one of the most speciose genera of snakes in the Western Hemisphere. Its geographic range extends from southwestern Chihuahua and southern Tamaulipas, Mexico, south and east through central and southern Mexico (except for the Yucatán Peninsula) and Central America to northern and western Colombia between 13 and 2744 m elevation (Downs 1967, Wilson and Townsend 2007).

**Figure 1.**
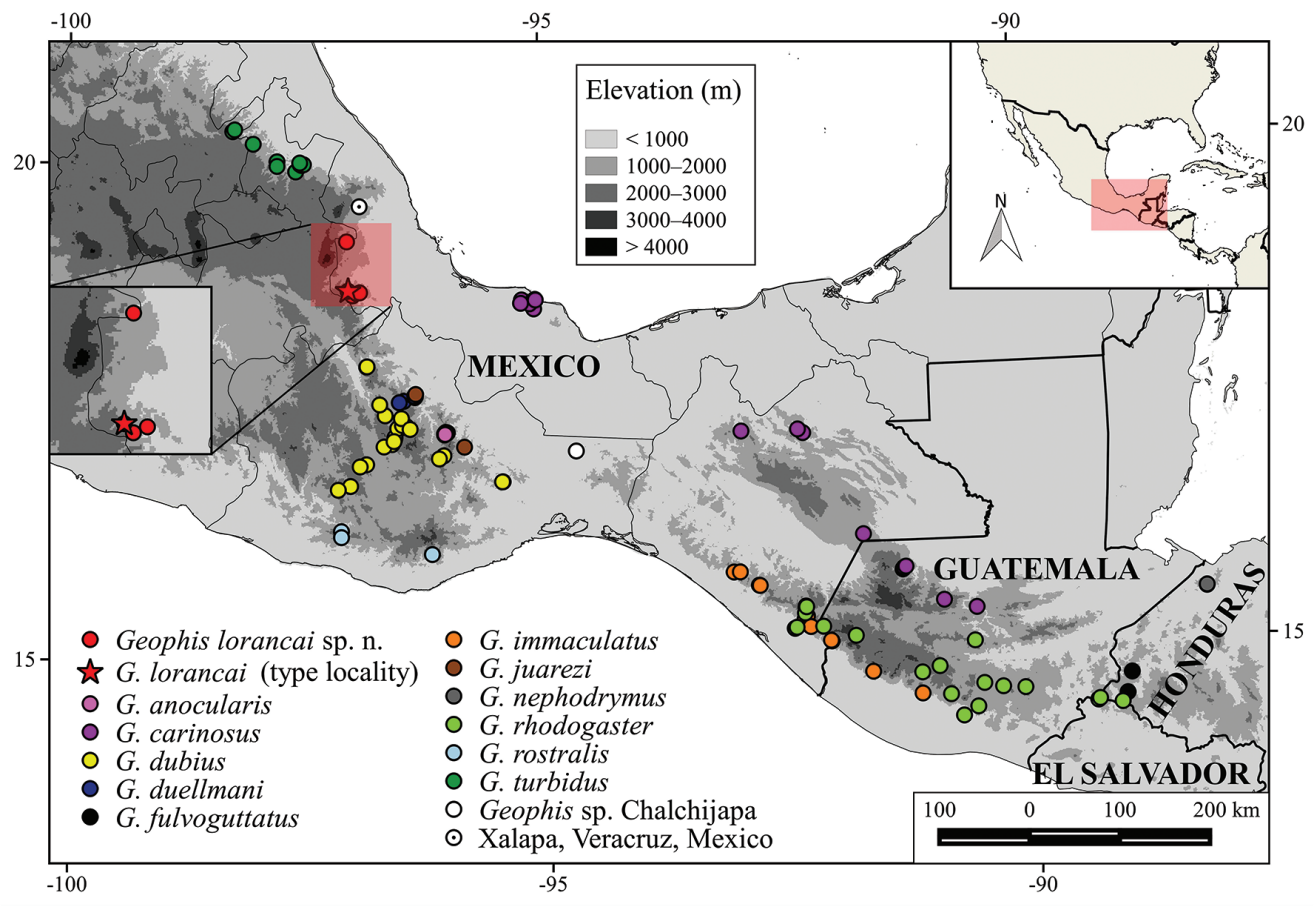
Distribution of the species of the *Geophis dubius* group. Bold lines represent country limits and narrow lines limits of Mexican states.

Downs (1967) divided *Geophis* into seven species groups (*chalybeus*, *championi*, *dubius*, *latifrontalis*, *omiltemanus*, *semidoliatus*, and *sieboldi*) mainly on the basis of their external morphology and dentition. The *dubius* group is currently composed of 11 species, which range collectively from northern Puebla and central Veracruz south and east through southeastern Mexico and Guatemala to El Salvador (Fig. 1): *Geophis anocularis*, from the Sierra Mixe in northern Oaxaca; *Geophis carinosus*, from the Sierra de Los Tuxtlas in southern Veracruz, the Atlantic slopes of Chiapas, and the Sierra de Los Cuchumatanes in Guatemala; *Geophis dubius*, from central and northern Oaxaca and perhaps central Veracruz; *Geophis duellmani*, from the Sierra de Juárez in northern Oaxaca; *Geophis fulvoguttatus*, from El Salvador and Honduras; *Geophis immaculatus*, from central Chiapas, Mexico, and the Pacific versant of Guatemala; *Geophis juarezi*, from the Sierra de Juárez and Sierra Mixe in northern Oaxaca; *Geophis nephodrymus*, from the Sierra de Omoa, Honduras; *Geophis rhodogaster*, which ranges from the mountains of southwestern Chiapas, Mexico, east through the western part of the Guatemalan Plateau and southeastern highlands of Guatemala to adjacent El Salvador and Honduras; *Geophis rostralis*, from southern Oaxaca, and *Geophis turbidus*, from northern Puebla (Downs 1967, Campbell et al. 1983, Smith 1995, Nieto-Montes de Oca 2003, Wilson and Townsend 2007, Townsend 2009, Pavón-Vázquez et al. 2013). Additionally, a specimen of uncertain status within the *Geophis dubius* group was reported by Nieto-Montes de Oca (2003) from the Chimalapas region, Oaxaca, Mexico. The *Geophis dubius* group was defined by Downs (1967) and redefined by Nieto-Montes de Oca (2003).

Most species of the *Geophis dubius* group are uniformly dark dorsally without conspicuous patterns, though a juvenile of *Geophis turbidus* exhibits a light collar. The only exceptions are *Geophis fulvoguttatus*, which has 17–22 irregular yellowish-brown to reddish blotches on the posterior part of the body and 3–4 irregular light blotches on the anterior half of the tail on a dark background (Downs 1967); *Geophis nephodrymus*, which ranges from patternless to extensively marked with bands, laterally offset partial bands, and lateral blotches that range from pale grayish cream to brick red on a gray dorsal background (Townsend 2009); and *Geophis duellmani*, which has a black head and anteriormost portion of the body and dark saddles on a red or white background on the rest of the body and tail (Campbell et al. 1983).

Herein, a new species of the *Geophis dubius* group is described with black cross-bands on an orange-red background color on most of the body and tail from the mountains of east-central Puebla and west-central Veracruz.

## Materials and methods

The sample of the new species (*n* = 8) was compared with specimens of all of the species of the *Geophis dubius* group from Mexico, with the exception of *Geophis rostralis*. A list of the specimens examined is provided in Suppl. material 1: Table 1. Acronyms for herpetological collections follow Sabaj Pérez (2014), with the addition of ITSZ (Instituto Tecnológico Superior de Zongolica). SMR is an abbreviation for field numbers of uncatalogued specimens in the MZFC.

Scale nomenclature follows Downs (1967) and Savage and Watling (2008). Scale counts were performed with the aid of a dissecting microscope. Ventrals were counted as suggested by Downs (1967). Bilateral characters were scored on both sides. When the condition of a given character was not identical on both sides, the conditions on the left and right sides are given, in that order, separated by a slash (/). Measurements were taken with a ruler, digital calipers, or an ocular micrometer to the nearest 0.1 mm. Head length was measured from the tip of the snout to the posterior end of the parietals. All scale dimensions were measured at their maximum. To examine dentition characters, the maxilla and ectopterygoid were removed from the skull and cleansed in a dilute solution of Proteinase K for approximately one hour. Color codes and descriptions follow Smithe (1975). The diagnosis is based on both the specimens examined and the relevant literature (Bogert and Porter 1966, Downs 1967, Smith and Holland 1969, Campbell and Murphy 1977, Savage 1981, Campbell et al. 1983, Restrepo and Wright 1987, Smith and Chiszar 1992, Smith and Flores-Villela 1993, Lips and Savage 1994, Smith 1995, Wilson et al. 1998, Pérez-Higareda et al. 2001, Myers 2003, Nieto-Montes de Oca 2003, Savage and Watling 2008, Townsend 2009, Townsend and Wilson 2006, Pavón-Vázquez et al. 2011, 2013).

## Results

### Geophis lorancai sp. n.

Taxon classificationAnimaliaSquamataDipsadidae

http://zoobank.org/364ED739-EBD8-4CC5-8124-B87C2E054AB3

Figs 2 , 3

#### Holotype

**Figure 2.**
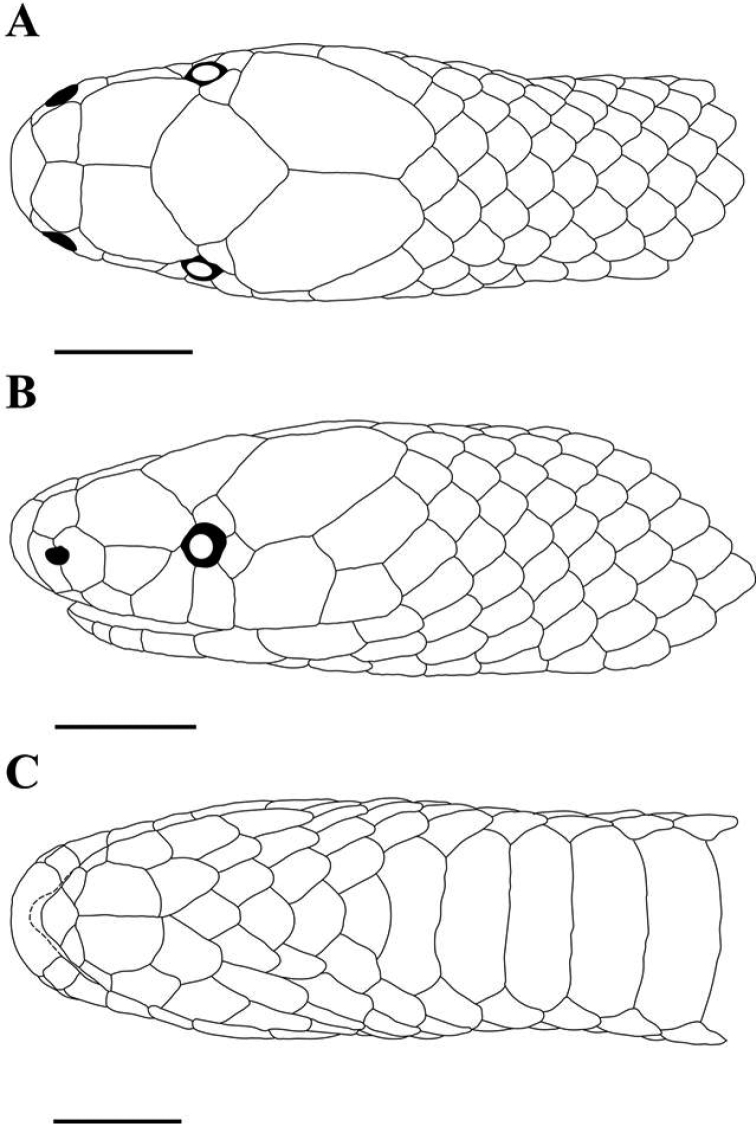
*Geophis lorancai*, holotype (MZFC 28401). Head scales in **A** dorsal **B** left lateral, and **C** ventral views. Horizontal lines = 3 mm.

(Fig. 2). MZFC 28401, an adult male from the Instituto Tecnológico Superior de Zongolica, vicinity of Atlanca, municipality of Los Reyes, Sierra de Zongolica, Veracruz, Mexico (18°41'48"N, 97°03'21"W), 1700 m elevation, collected by Miguel Angel de la Torre Loranca on April 6, 2008.

#### Paratypes.

Seven specimens, six from the Sierra de Zongolica of west-central Veracruz and one from the Sierra de Quimixtlán of adjacent Puebla, Mexico. Veracruz: Three from the same locality as the holotype (MZFC 28402–03, ITSZ 217); one from Zongolica, 18°39'02"N, 97°00'29"W, 1210 m (ITSZ 071); one from 7 km E Zongolica (MZFC 28405); one from Los Reyes, 18°41'48"N, 97°03'21"W, 1700 m (ITSZ 025). Puebla: Chichiquila, 19°11'35"N, 97°03'57"W, 1700 m (MZFC 28404).

#### Diagnosis.

A member of the *Geophis dubius* group characterized by the following combination of traits: eye relatively small (see below); single supraocular and postocular present on each side; fifth supralabial and parietal in contact; mental scale and anterior chinshields in contact; smooth dorsal scales throughout the body arranged in 17 rows; ventrals 130, *n* = 1, in females, and 125–130, *n* = 7, in males; subcaudals in males 33–35, *n* = 5; dorsal body and tail pattern consisting of dark crossbands on a paler, red-orange background; reddish orange venter; maxillary teeth 7.

*Geophis lorancai* may be distinguished from all of the species in the *championi* and *semidoliatus* groups, and all of the species in the *sieboldi* group except *Geophis dunni*, *Geophis nasalis*, *Geophis occabus*, and *Geophis sieboldi* by having the dorsal scales arranged in 17 rows (versus dorsal scales arranged in 15 rows in the other species); and from the latter four species by having smooth dorsal scales throughout the body (versus dorsal scales keeled on at least the posterior half of the body in the other species).

*Geophis lorancai* differs from all of the species in the *omiltemanus* and *chalybeus* groups by having a small eye (i.e., its horizontal diameter contained nearly four times in the snout length, versus its horizontal diameter contained less than three times in the snout length in the other species); in addition, it may be distinguished from all of the species in the *omiltemanus* group by having the fifth supralabial and parietal in contact (versus fifth supralabial and parietal separated by one anterior temporal in the other species); from some species in the *chalybeus* group (*Geophis dugesii*, *Geophis nigrocinctus*, and *Geophis tarascae*) by having the dorsals arranged in 17 rows (versus dorsals arranged in 15 rows in the other species); and from the remaining species in this group (*Geophis bicolor* and *Geophis chalybeus*) by having the mental and anterior chinshields in contact (versus mental and anterior chinshields separated by the first pair of infralabials in the other species).

*Geophis lorancai* may be distinguished from the species in the *latifrontalis* group as follows: from *Geophis blanchardi* and *Geophis mutitorques*, by having a dorsal body pattern consisting of dark crossbands on a paler, red-orange background (versus dorsum uniformly dark in *Geophis blanchardi* and adults of *Geophis mutitorques*—juveniles with yellow collar); from *Geophis latifrontalis* and *Geophis mutitorques*, by having the fifth supralabial and parietal in contact (versus fifth supralabial and parietal separated by one anterior temporal in *Geophis latifrontalis* and *Geophis mutitorques*); and from *Geophis blanchardi* and *Geophis latifrontalis*, by having the mental and anterior chinshields in contact (versus mental and anterior chinshields separated by the first pair of infralabials in *Geophis blanchardi* and *Geophis latifrontalis*).

*Geophis lorancai* may be distinguished from the species in the *dubius* group as follows (Suppl. material 2: Table 2): from *Geophis anocularis*, *Geophis carinosus*, *Geophis dubius*, *Geophis immaculatus*, *Geophis juarezi*, *Geophis rhodogaster*, *Geophis rostralis*, and *Geophis turbidus* by having a dorsal body and tail pattern consisting of dark crossbands on a paler, red-orange background (versus dorsum uniformly dark in the other species, except for a pink collar present in a juvenile of *Geophis turbidus*); from *Geophis duellmani* by having one supraocular and one postocular (versus supraocular and postocular absent in *Geophis duellmani*); from *Geophis fulvoguttatus* by having fewer ventrals (130, *n* = 1, in females, and 125–130, *n* = 7, in males; versus 145–147, *n* = 2, in females, and 135–137, *n* = 2, in males of *Geophis fulvoguttatus*); and from *Geophis nephodrymus* by having fewer maxillary teeth (7, *n* = 3; versus 11, *n* = 1, in *Geophis nephodrymus*), a reddish orange venter (versus venter predominantly gray or yellowish cream in *Geophis nephodrymus*) and more subcaudals in males (33–35, *n* = 5; versus 22–31, *n* = 6, in *Geophis nephodrymus*).

#### Description of holotype

(Fig. 2). Adult male. Head length = 8.9 mm, snout-vent length (SVL) = 268 mm, tail length = 53.4 mm. Head slightly distinct from body. Snout long, contained 2.2 times in head, rounded from above, projecting anteriorly far beyond lower jaw; rostral 0.7 times as broad as high, portion visible from above 0.3 times as long as its distance from frontal, 1.2 times as long as internasals common suture, with posterior end approximately at level of anterior margin of nostrils; internasals broader than long (length / breadth= 0.7/0.8), slightly angulate anteriorly, in lateral contact with anterior and posterior nasals, their length and common suture 0.7/0.8 and 0.5 times as long as prefrontals common suture, respectively. Prefrontals in lateral contact with postnasal, loreal, and eye on each side, and additionally with third supralabial on left side; their length 0.6 times snout length; their common suture 0.5 times frontal length. Frontal slightly broader than long (breadth / length ratio = 1.1). Supraocular large, in contact with prefrontal, frontal, parietal, and postocular; approximately 0.9 times as long as horizontal diameter of eye, 0.6 times as long as loreal, bordering posterior half of dorsal margin of orbit, ventral margin slightly projecting posteriorly beyond posterior margin of orbit. Parietals 1.5/1.6 times as long as broad, their length 0.5 times head length, their common suture 0.8 times as long as frontal.

Nasal divided. Postnasal 1.3/1.1 times as long as prenasal. Combined length of prenasal and postnasal subequal to loreal length. Loreal 1.4/1.5 times as long as deep, contained 2.7/2.5 times in snout length, 1.6 times as long as horizontal diameter of eye, its dorsal margin slightly concave; failing to reach orbit on the left side, in broad contact with anterior margin of orbit on right side. Eye small, contained 4.3 times in snout length, its vertical diameter 0.7 times its distance from lip. One postocular, 1.4/1.3 times as high as long, 1/0.8 times as long as supraocular. Supralabials 6/6, first and second in contact with postnasal, second and third in contact with loreal, third and fourth entering orbit (third contacting prefrontal on left side), fifth largest, in contact with parietal. Ventral margin of third supralabial 1.2/1.6 times that of second supralabial; ventral margin of fifth supralabial 1.9 times that of fourth supralabial, 1.0/1.1 times that of sixth supralabial. Anterior temporal absent. One posterior temporal. Five nuchal scales in contact with parietals.

Mental 1.2 times as broad as long, pointed anteriorly, in posterior contact with anterior chinshields. Infralabials 6/6, first to third in contact with anterior chinshields, third and fourth in contact with posterior chinshields. Anterior chinshields 1.6/1.7 times as long as broad, 1.4 times as long as posterior chinshields. Posterior chinshields in narrow contact with each other anteriorly, separated posteriorly by one midgular scale. Four midgular scales. Infralabials and scales in chin region smooth. Dorsals in 17-17-17 rows, smooth throughout the body; no evident apical pits. Ventrals 128. Subcloacal scute single. Paired subcaudals 34.

**Figure 3.**
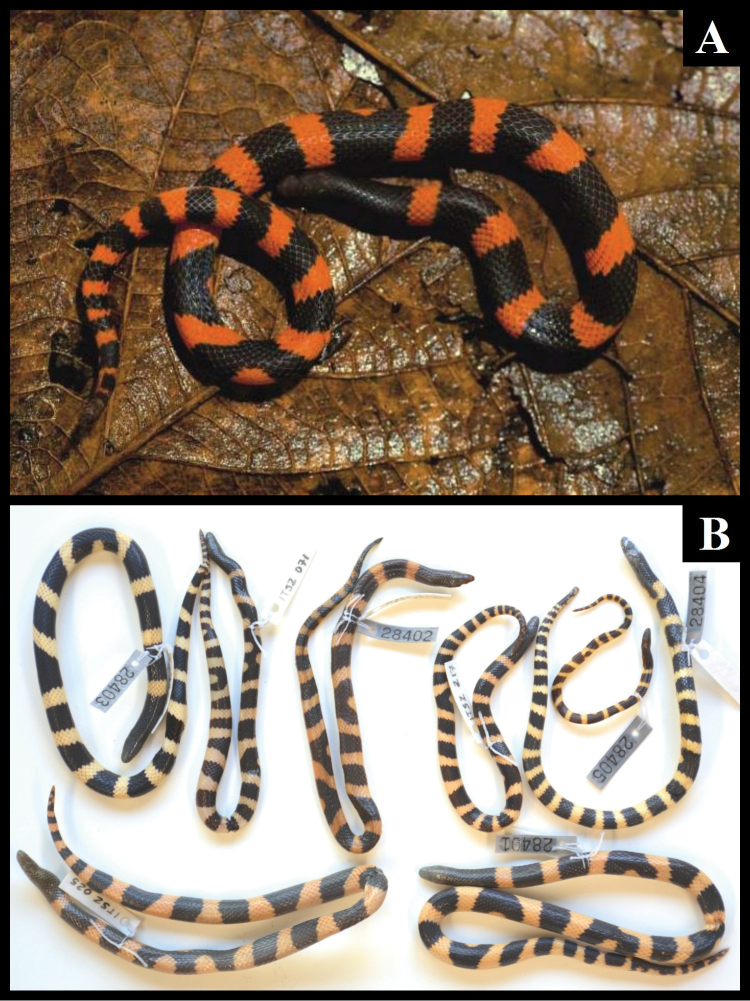
Coloration in *Geophis lorancai*: **A** adult male paratype (MZFC 28402), photo by Miguel Ángel de la Torre Loranca; and **B** type series.

*Color in life* (Fig. 3): Dorsal and lateral surfaces of head and anterior end of body (to approximately level of 12^th^ middorsal scale) black; those of rest of body and tail with black transverse marks on a reddish orange background color (color 17, Spectrum orange; Smithe, 1975). Fifteen black transverse marks on body; 1^st^–2^nd^, 4^th^–5^th^, 7^th^, 9^th^, 11^th^–12^th^, and 14^th^–15^th^ saddle-shaped, about 6–10 scales in length, usually extending laterally to lateral margin of ventral scales (one saddle extending laterally to third dorsal row on right side); remaining five marks consisting each of a transverse band extending on one side of body and bifurcating at midline into two transverse bands on opposite side, forming a Y-shaped mark; Y-shaped marks 7–9 scales in length at their longest. Reddish orange rings between saddles 4–6 scales in length. Eight black transverse marks on tail; anterior three saddle-shaped, about 1.5–4 scales in length, extending laterally to lateral margin of subcaudals or nearly so; 4^th^ mark irregular, elongate, presumably formed by fusion of several adjacent transverse marks, 9.5 scales in length at its longest, in lateral contact with first dorsal scale row at three points on each side; remaining marks shorter, irregular; posterior end of tail with black and red checkered pattern. Reddish orange rings between black marks 1–2.5 scales in length. Ventral surface of head pale grey; that of body and tail immaculate reddish orange except for one dark splotch on middle of 7^th^ ventral and black lateral ends of those ventrals involved in transverse body marks; tail surface increasingly dark posteriorly.

#### Dentition.

The description below is based on the dentition on the right side of paratypes ITSZ 25 and MZFC 28402 and on the left side of paratype MZFC 28403. Scored characters were consistent in all the specimens. Maxilla extending anteriorly to level of suture between 2^nd^ and 3^rd^ supralabials; posterior fourth of maxilla curved ventrally in lateral view; anterior tip of maxilla toothless, bluntly pointed; maxillary teeth 7, recurved; teeth slightly longer at middle of maxilla; large flange projecting medially at approximately level of middle of maxilla; posterior end of maxilla laterally compressed into moderate flange. Anterior end of ectopterygoid bifurcate; dorsal branch long, compressed; ventral branch very short, stump-like.

#### Variation.

This section is based on the examination of the seven paratypes. We describe only character conditions that differ from those in the holotype. Ranges are given for some characters. When ranges included the holotype, we report its condition. Two males (MZFC 28403–28404) have an incomplete tail; thus, subcaudal and total segmental counts, tail length, tail length / total length ratio, and number of tail black markings are not reported for those specimens.

Posterior temporal divided transversally on both sides in three specimens (ITSZ 71, MZFC 28402, MZFC 28405); nuchal scales in contact with parietals between posterior temporals 3–7, x = 5.7 (3, in one specimen; 5, in two; 6, in one; 7, in three with transversally divided posterior temporals). Second and third supralabials fused on both sides in ITSZ 25 (supralabials 5/5); infralabials 7/7 in one specimen (ITSZ 71). Posterior chinshields in medial contact with each other in four specimens, separated by one midgular scale in three. Midgular scales 2–3, x = 2.9 (2, in one specimen; 3, in six). Ventrals 130 in single known female; 125–130, x = 127.7, *n* = 7, in males, including holotype (one small scale anterior to subcloacal scute, not reaching lateral-most row of dorsals on one side, excluded from ventral counts in ITSZ 71 and MZFC 28402). Subcaudals 25 in single known female, 33–35, x = 34, *n* = 5, in males, including holotype; total segmental counts 155 in single known female, 159–165, x = 162.2, *n* = 5, in males, including holotype. Apical pits evident over vent region in two specimens (ITSZ 217, MZFC 28403).

*Color pattern* (Fig. 3). General pattern similar to that of holotype in all specimens. Transverse dark marks on body and tail 13–20 and 4–10, respectively. Body pattern composed of dark crossbands and Y-shaped marks in all specimens (10 and 2, in ITSZ 25; 13 and 4, in ITSZ 71; 17 and 3, in ITSZ 217; 12 and 4, in MZFC 28402; 12 and 2, in MZFC 28403; 18 and 1, in MZFC 28404; 15 and 2, in MZFC 28405) and single, unusual marks in some specimens: adjacent transverse bands connected to each other, forming a zigzagging mark, in two specimens (zigzagging mark in contact with lateral edge of ventral scales at three points on each side in ITSZ 25, at four points on one side and three points on opposite side in ITSZ 71); two adjacent transverse bands connected along midline, forming a H-shaped mark, in one specimen (MZFC 28404). Tail pattern composed of black crossbands in all specimens (7, in ITSZ 25; 8, in ITSZ 71; 2, in ITSZ 217; 7, in MZFC 28402; 5, in MZFC 28405) in addition to one Y-shaped mark in ITSZ 71, and one zigzagging mark in contact with lateral edge of ventral scales at two points on one side and three points on opposite side in ITSZ 217; posterior end of tail light in ITSZ 25, dark in MZFC 28402 and MZFC 28405, and checkered with red and black in ITSZ 71 and ITSZ 217. Ventral surface of head and body immaculate, except for black stippling, heavier on anterior portion of body, in ITSZ 71, and dark first ventral, lateral ends of ventrals gradually paler posteriorly, in two specimens (MZFC 28402–28403); ventral surface of tail paler, subcaudals adjacent to black dorsal marks with dark lateral ends; dark pigment gradually heavier posteriorly; dark pigment on posterior edge of most subcaudals in two specimens (MZFC 28402 and 28404).

#### Etymology.

The specific name is treated as a noun in the genitive case and honors Biologist Miguel Ángel de la Torre Loranca, who obtained most of the specimens of the new species from the Sierra de Zongolica.

#### Distribution and ecology

(Fig. 1). *Geophis lorancai* is known from the Sierra de Zongolica of west-central Veracruz and the Sierra de Quimixtlán in adjacent extreme east-central Puebla between 1210 and 1700 m elevation (Fig. 1). All of the specimens of *Geophis lorancai* were obtained between October 1996 and April 2013. In these sierras, the terrain is irregular with numerous hills (some of them isolated), ascents and descents, and streams. The terrain shows a general decline towards the Gulf coastal plain (i.e., from west to east). The area is covered with cloud forest and pine-oak associations. In both sierras, *Geophis lorancai* is sympatric with *Geophis semidoliatus*, another species with dark crossbands on a red background belonging to the *Geophis semidoliatus* group.

All of the specimens of *Geophis lorancai* were found in cloud forest. The principal arboreal components of the vegetation at the type locality are *Liquidambar styraciflua*, *Quercus* spp., *Saurauia scabrida*, *Clethra mexicana*, *Lippia myriocephala*, *Heliocarpus appendiculatus*, *Magnolia mexicana*, *Carpinus carolineana*, and *Ternstroemia sylvativa*. The bush stratum is dominated by *Psychotria galeottiana*, *Piper* ssp., *Phyllonoma laticuspis*, and *Miconia* spp. Species found in the herbaceous stratum are *Smilax* spp., *Selaginella* spp., *Begonia* spp., *Monstera deliciosa*, *Philodendron* spp., *Salvia* spp., and *Dhalia coccinea*. Epiphytes of the Bromeliaceae and Orchidaceae families are common in this type of vegetation and mainly represented by the following species: *Tillandsia punctulata*, *Tillandsia multicaulis*, *Nidema boothii*, *Lycaste deppei*, and *Lycaste consobrina* (Rzedowski 1978, Cázares Hernández personal communication). The specimens of *Geophis lorancai* were found either among the leaf litter or under fallen logs.

## Discussion

With the addition of *Geophis lorancai*, the number of species in the genus increases to 50, and the number of species in the *Geophis dubius* group to 12, of which ten occur in Mexico (Fig. 1): seven are endemic to Mexico (*G anocularis*, *Geophis dubius*, *Geophis duellmani*, *Geophis juarezi*, *Geophis lorancai*, *Geophis rostralis*, and *Geophis turbidus*), two are shared only with Guatemala (*Geophis carinosus* and *Geophis immaculatus*), and one with Guatemala, El Salvador, and Honduras (*Geophis rhodogaster*). Two species are distributed only in Central America: *Geophis nephodrymus* in Honduras and *Geophis fulvoguttatus* in Honduras and El Salvador.

*Geophis lorancai* fills a gap in the distribution of the *Geophis dubius* group between the ranges of *Geophis turbidus* and those of the species from northern Oaxaca. Pavón-Vázquez et al. (2013) described *Geophis turbidus* from northern Puebla. This species has been recently reported also from adjacent Hidalgo (Cruz-Elizalde et al. 2015), and represents the northern limit of the distribution of the *Geophis dubius* group. With the exception of a doubtful record of *Geophis dubius* from “Jalapa” in central Veracruz (see below), the closest records to *Geophis lorancai* of this group are found in northern Oaxaca, where several endemic species are found (i.e., *Geophis anocularis*, *Geophis duellmani*, and *Geophis juarezi*). Other species found in Oaxaca are *Geophis dubius*, widely distributed in central and northern Oaxaca, and *Geophis rostralis*, from the Sierra Madre del Sur.

The validity of the record from Jalapa has been questioned. Bogert and Porter (1966) considered this record as doubtful on the basis that although “Jalapa” was the locality given by Fischer (1886) as the source of the specimen he described as *Geophis fuscus*, he did not specify the state and there are ‘‘at least a dozen’’ other localities with the same name in Mexico, including two in the state of Oaxaca. For many years central Veracruz has been a collecting site for many herpetologists, yet *Geophis dubius* has never been found there. The only species of *Geophis* found in central Veracruz are the common *Geophis semidoliatus* (*semidoliatus* group), *Geophis blanchardi*, *Geophis mutitorques* (both belonging to the *latifrontalis* group), and *Geophis chalybeus* (*chalybeus* group). According to this, and the fact that *Geophis dubius* is a common species in the state of Oaxaca, it seems possible that the specimen from Jalapa assigned to this species comes from Oaxaca.

*Geophis lorancai* and *Geophis turbidus* are similar in scalation and relatively close geographically. Thus, it is conceivable that these two taxa represent the same species with two color morphs: one predominantly dark and another one with dark crossbands on a red-orange background, as in the polymorphic species *Geophis occabus* and *Geophis brachycephalus* (Pavón-Vázquez et al. 2011). However, a phylogenetic analysis of *Geophis* based on several mitochondrial and nuclear genes shows that *Geophis lorancai* and *Geophis turbidus* are considerably divergent genetically and not each other’s sister species (Canseco-Márquez et al. unpublished data).

### Key to the species of the *Geophis dubius* group

**Table:** 

1	Dorsum uniformly dark—pink collar may be present	**2**
–	Dorsum bearing reddish or whitish bands, blotches, or saddles	**10**
2	Dorsal scales keeled on at least posterior half of body	**3**
–	Dorsal scales smooth throughout body or, if keeled, keeling restricted to posterior fourth of body or less	**4**
3	Supraocular in broad contact with frontal; posterior chinshields in broad medial contact anteriorly; ventrals 116–123 in males, 125–136 in females	***Geophis carinosus***
–	Supaocular separated from or in narrow contact with frontal; posterior chinshields usually separated or in narrow medial contact; ventrals 114–115 in males, 118–124 in females	***Geophis juarezi***
4	Supraocular absent	**5**
–	Supraocular usually distinct or, if indistinct, indistinctness caused by the obvious fusion with another scale	**6**
5	Postocular absent	***Geophis anocularis***
–	Postocular present	***Geophis rhodogaster***
6	Maxillary teeth usually fewer than 12, anterior tip of maxilla toothless (not from the Sierra Madre of Guatemala and Chiapas, Mexico)	**7**
–	Maxillary teeth 12, anteriormost maxillary tooth born at anterior tip of maxilla (Sierra Madre of Guatemala and Chiapas, Mexico)	***Geophis immaculatus***
7	Ventrals + subcaudals more than 160 (159 in a single female of *Geophis turbidus* from northern Puebla, Mexico)	**8**
–	Ventrals + subcaudals 160 or less (Sierra de Omoa, Honduras)	***Geophis nephodrymus*** (in part)
8	Internasals and prefrontals usually fused; loreal longer than combined prenasal and postnasal length; 131 ventrals or more	***Geophis dubius***
–	Not as above	**9**
9	Subcaudals 40 or more; tail length / total length ratio 0.20 or more (Sierra Madre del Sur, Oaxaca, Mexico)	***Geophis rostralis***
–	Subcaudals fewer than 40; tail length / total length ratio 0.18 or less (Sierra Madre Oriental in Puebla and Hidalgo, Mexico)	***Geophis turbidus***
10	Supraocular absent	***Geophis duellmani***
–	Supraocular usually distinct or, if indistinct, indistinctness caused by the obvious fusion with another scale	**11**
11	Ventrals + subcaudals less than 171	**12**
–	Ventrals + subcaudals 171 or more	***Geophis fulvoguttatus***
12	Maxillary teeth 7; ventral surface of body reddish orange; subcaudals 33–35 in males (Sierra de Quimixtlán, Puebla, and Sierra de Zongolica, Veracruz, Mexico)	***Geophis lorancai***
–	Maxillary teeth 11; ventral surface of body predominantly gray or yellowish cream; subcaudals 22–31 in males (Sierra de Omoa, Honduras)	***Geophis nephodrymus* (in part)**

## Supplementary Material

XML Treatment for Geophis lorancai

## Supplementary materials

Supplementary material 1 Table S1. Specimens examined. Data type: MS Word file Explanation note: All of the specimens are from Mexico. The table is arranged alphabetically by species name and specimen voucher number, in that order. This dataset is made available under the Open Database License (http://opendatacommons.org/licenses/odbl/1.0/). The Open Database License (ODbL) is a license agreement intended to allow users to freely share, modify, and use this Dataset while maintaining this same freedom for others, provided that the original source and author(s) are credited.Luis Canseco-Márquez, Carlos J. Pavón-Vázquez, Marco Antonio López-Luna, Adrián Nieto-Montes de Oca

Supplementary material 2 Table 2. Selected characters in the species of the *Geophis dubius* group. Data type: MS Word file This dataset is made available under the Open Database License (http://opendatacommons.org/licenses/odbl/1.0/). The Open Database License (ODbL) is a license agreement intended to allow users to freely share, modify, and use this Dataset while maintaining this same freedom for others, provided that the original source and author(s) are credited.Luis Canseco-Márquez, Carlos J. Pavón-Vázquez, Marco Antonio López-Luna, Adrián Nieto-Montes de Oca

## References

[B1] Bogert CM Porter AP (1966) The differential characteristics of the Mexican snakes related to *Geophis dubius* (Peters). American Museum Novitates 2277: 1–19.

[B2] Campbell JA Murphy JB (1977) A new species of *Geophis* (Reptilia, Serpentes, Colubridae) from the Sierra de Coalcoman, Michoacan, Mexico. Journal of Herpetology 11: 397–403. doi: 10.2307/1562721

[B3] Campbell JA Ford LS Karges JP (1983) Resurrection of *Geophis anocularis* Dunn with comments on its relationships and natural history. Transactions of the Kansas Academy of Sciences 86: 38–47. doi: 10.2307/3628422

[B4] Cruz-Elizalde R Ramírez-Bautista A Lara-Trufiño D (2015) New record of the snake *Geophis turbidus* (Squamata: Dipsadidae) from Hidalgo, Mexico, with annotations of a juvenile specimen. Check List 11: . doi: 10.15560/11.4.1724

[B5] Downs FL (1967) Intrageneric relationships among colubrid snakes of the genus *Geophis* Wagler. Miscellaneous Publications of the Museum of Zoology, University of Michigan 131: 1–193.

[B6] Lips KR Savage JM (1994) A new fossorial snake of the genus *Geophis* (Reptilia: Serpentes: Colubridae) from the Cordillera de Talamanca of Costa Rica. Proceedings of the Biological Society of Washington 107: 410–416.

[B7] Myers CW (2003) Rare snakes—five new species from eastern Panama: reviews of northern *Atractus* and southern *Geophis* (Colubridae: Dipsadinae). American Museum Novitates 3391: 1–47. doi: 10.1206/0003-0082(2003)391<0001:RSFNSF>2.0.CO;2

[B8] Nieto-Montes de Oca A (2003) A new species of the *Geophis dubius* group (Squamata: Colubridae) from the Sierra de Juárez of Oaxaca, Mexico. Herpetologica 59: 572–585. doi: 10.1655/02-05

[B9] Pavón-Vázquez CJ Canseco-Márquez L Nieto-Montes de Oca A (2013) A new species in the *Geophis dubius* group (Squamata: Colubridae) from northern Puebla, México. Herpetologica 69: 358–370. doi: 10.1655/HERPETOLOGICA-D-12-00095

[B10] Pavón-Vázquez CJ García-Vázquez UO Blancas-Hernández JC Nieto-Montes de Oca A (2011) A new species of the *Geophis sieboldi* group (Squamata: Colubridae) exhibiting color pattern polymorphism from Guerrero, Mexico. Herpetologica 67: 332–343. doi: 10.1655/HERPETOLOGICA-D-11-00003.1

[B11] Pérez-Higareda G Smith HM López-Luna MA (2001) A new *Geophis* (Reptilia: Serpentes) from southern Veracruz, Mexico. Bulletin of the Maryland Herpetological Society 37: 42–48.

[B12] Restrepo TJH Wright JW (1987) A new species of the colubrid snake genus *Geophis* from Colombia. Journal of Herpetology 21: 191–196. doi: 10.2307/1564482

[B13] Rzedowski J (1978) Vegetación de México. Limusa, Ciudad de México, 432 pp.

[B14] Sabaj Pérez MH (Ed.) (2014) Standard symbolic codes for institutional resource collections in herpetology and ichthyology: an Online Reference. Version 5.0 (22 September 2014). Available at http://asih.org/. American Society of Ichthyologists and Herpetologists, Washington, DC.

[B15] Savage JM (1981) A new species of the secretive colubrid snake genus *Geophis* from Costa Rica. Copeia 1981: 549–553. doi: 10.2307/1444557

[B16] Savage JM Watling JI (2008) Not so rare snakes: a revision of the *Geophis sieboldi* group (Colubridae:Dipsadinae) in lower Central America and Colombia. Zoological Journal of the Linnean Society 153: 561–599. doi: 10.1111/j.1096-3642.2008.00400.x

[B17] Smith EN (1995) *Geophis rhodogaster* (Colubridae), an addition to the snake fauna of Mexico. The Southwestern Naturalist 40: 123–124.

[B18] Smith HM Chiszar D (1992) A second locality for *Geophis sallei* (Reptilia: Serpentes). Bulletin of the Maryland Herpetological Society 28: 16–18.

[B19] Smith HM Flores-Villela O (1993) Variation in two species (*Geophis* bicolor, *Geophis duellmani*) of Mexican earth snakes (*Geophis*). Bulletin of the Maryland Herpetological Society 29: 20–23.

[B20] Smith HM Holland RL (1969) Two new snakes of the genus *Geophis* from Mexico. Transactions of the Kansas Academy of Sciences 72: 47–53. doi: 10.2307/3627047

[B21] Smithe FB (1975) Naturalist’s Color Guide. American Museum of Natural History, New York, 229 pp.

[B22] Townsend JH (2006) Inventory and conservation assessment of the herpetofauna of the Sierra de Omoa, Honduras, with a review of the *Geophis* (Squamata: Colubridae) of eastern Nuclear Central America. Master’s Degree Thesis, University of Florida, Miami, USA.

[B23] Townsend JH (2009) Morphological variation in *Geophis nephodrymus* (Squamata: Colubridae), with comments on conservation of *Geophis* in eastern Nuclear Central America. Herpetologica 65: 292–302. doi: 10.1655/07-039R2.1

[B24] Townsend JH Wilson LD (2006) A new species of snake of the *Geophis dubius* group (Reptilia: Squamata: Colubridae) from the Sierra de Omoa of northwestern Honduras. Proceedings of the Biological Society of Washington 119: 150–159. doi: 10.2988/0006-324X(2006)119[150:ANSOSO]2.0.CO;2

[B25] Wilson LD McCranie JR Williams KL (1998) A new species of *Geophis* of the *sieboldi* group (Reptilia:Squamata: Colubridae) from northern Honduras. Proceedings of the Biological Society of Washington 111: 410–417.

[B26] Wilson LD Townsend JH (2007) A checklist and key to the snakes of the genus *Geophis* (Squamata: Colubridae: Dipsadinae), with commentary on distribution and conservation. Zootaxa 1395: 1–31.

