# Supplementary material for: A new species of earth snake (Dipsadidae, Geophis) from Mexico
Source: Zookeys. 2016 Aug 11;(610):131–45. doi: 10.3897/zookeys.610.8605 (PMC4992814; doi:10.3897/zookeys.610.8605)
Supplement: Supplementary material 1 — Table S1. Specimens examined. [file zookeys-610-131-s001.doc]

**Table 1.** Specimens examined. All of the specimens are from Mexico. The table is arranged alphabetically by species name and specimen voucher number, in that order.

| Species | State | Locality | Latitude (if available) | Longitude (if available) | Specimen vouchers |
| --- | --- | --- | --- | --- | --- |
| *Geophis anocularis* | Oaxaca | Sierra Mixe, 1.2 mi W Totontepec | – | – | MZFC 16178 |
|  |  | 5 km S Totontepec | – | – | MZFC 11591 |
|  |  | Sierra Mixe, 1.8 mi W Totontepec | – | – | MZFC 16180 |
| *G. carinosus* | Veracruz | SW slope of Volcan San Martín, Rancho Primero de Mayo, approximately 12.3 km NE Tapalapan | 18°32’55”N | 96°14’11”W | MZFC 10552 |
| *G. dubius* | Oaxaca | 2.6–3.1 km E La Cumbre, road to Yuvila | 16°55’48”N | 96°54’36”W | CNAR 6732 |
|  |  | Sierra Monteflor, near Arroyo el Chorro | – | – | EBUAP 1966 |
|  |  | Xiacui, agroforestral area of the town | – | – | MZFC 13887 |
|  |  | Cerro San Felipe | 17°13’44”N | 96°43’37”W | MZFC 16160, 16193 |
|  |  | Road Santa María Guienagati-Lachidola, north slope of Cerro Las Flores | 16°45’39”N | 95°28’6”W | MZFC 16547 |
|  |  | 4 km SSW San Isidro Buenos Aires | 17°55’56”N | 96°52’6”W | MZFC 27255 |
|  |  | 4 km SE San Isidro Buenos Aires | 17°56’4”N | 96°51’52”W | MZFC 27256 |
|  |  | Ca. 2 km from the deviation to San Martín Buenavista, coming from San Pedro Yolox | 17°36’27”N | 96°32’28”W | MZFC 27257 |
|  |  | Ixtlán de Juárez | – | – | MZFC 27258 |
| *G. duellmani* | Oaxaca | La Esperanza | – | – | MZFC 4524–4525 |
|  |  | Cerro Machín | – | – | MZFC 4526 |
|  |  | Sierra de Juárez, km 88 | – | – | MZFC 4527 |
|  |  | La Esperanza, km 80.5 | – | – | MZFC 5081 |
| *G. immaculatus* | Chiapas | El Triunfo | – | – | MZFC 27259 |
|  |  | Municipality of Motozintla de Mendoza, 0.7 km S (by air) of Ejido Boquerón | 15°14’0”N | 92°17’44”W | MZFC 27260 |
| *G. juarezi* | Oaxaca | Municipality of Santiago Comaltepec, vicinity of Metates, Sierra de Juárez | 17°39’3”N | 96°21’26”W | MZFC 2236 (holotype) |
|  |  | Municipality of Santa María Alotepec, 2.39 km N of Santa María Alotepec | 17°6’50”N | 95°51’9”W | MZFC 27525 |
| *G. rhodogaster* | Chiapas | Cerro Boqueron, 1.1 km WSW (by air) of Ejido Boqueron | – | – | SMR 1873 |
|  |  | Cerro Boqueron, 0.8 km WSW (by air) of Ejido Boqueron | – | – | SMR 1847 |
|  |  | 1.8 km NE (by rd) of summit of Cerro Mozotal on road to Motozintla | – | – | SMR 1831 |
| *G. turbidus* | Puebla | Municipality of Tepango de Rodríguez, Tepango de Rodríguez | – | – | CNAR 6886–6888 |
|  |  | Municipality of Tepango de Rodríguez, 5 km S Tepango de Rodríguez | – | – | CNAR 6889 |
|  |  | 3 km N Zacapoaxtla, road to Cuetzalan | – | – | CNAR 8233 |
|  |  | municipality of Cuetzalan, 5.5 km SSW Cuetzalan | – | – | EPUAP 521 |
|  |  | municipality of Cuetzalan, 2 km NW Xocoyolo, near Vista Hermosa | 19°58’41”N | 97°32’18”W | EBUAP 1021 |
|  |  | Municipality of Cuetzalan, La Loma del Chivo, on the road to Tacopixacta | 19°58’25”N | 97°30’59”W | EBUAP 1022 |
|  |  | 16.09 km SW Villa Juárez, also known as Xicotepec de Juárez | – | – | KU 39642 |
|  |  | Municipality of Cuetzalan, hillside in Xocoyolo, on road to Apulco River | 19º57’57.6”N | 97º32’13.1”W | MZFC 27253 |
|  |  | municipality of Cuetzalan, 3.5 km W Xocoyolo | 19º59’26”N | 97º33’20”W | MZFC 27254 (holotype) |
