# Supplementary material for: A new species of earth snake (Dipsadidae, Geophis) from Mexico
Source: Zookeys. 2016 Aug 11;(610):131–45. doi: 10.3897/zookeys.610.8605 (PMC4992814; doi:10.3897/zookeys.610.8605)
Supplement: Supplementary material 2 — Table 2. Selected characters in the species of the Geophis dubius group. [file zookeys-610-131-s002.doc]

Table 2. Selected characters in the species of the *G. dubius* group.

*Footnotes*

† Data obtained from examined specimens

‡ Campbell et al., 1983

§ Townsend, 2009

| Nieto-Montes de Oca, 2003

¶ Downs, 1967

# Smith and Flores-Villela, 1993

†† Smith and Holland, 1969

§§ Townsend, 2006

|| Pavón-Vázquez et al., 2013

¶¶ Cruz-Elizalde et al., 2015

|  | ***G. anocularis* ^†, ‡, §^** | ***G. carinosus* |, ¶, §** | ***G. dubius* ^†, ‡, §^** | ***G. duellmani* ^‡, #, ††^** | ***G. fulvoguttatus* ^¶, §, §§^** | ***G. immaculatus* ^†, ¶^** | ***G. juarezi |*** | ***G. lorancai* ^†^** | ***G. nephodrymus* ^§, §§^** | ***G. rhodogaster* ^†, ¶, §, §§^** | ***G. rostralis* ^‡, §^** | ***G. turbidus* ||, ¶¶** | ***G.* sp. Chalchijapa |** |
| --- | --- | --- | --- | --- | --- | --- | --- | --- | --- | --- | --- | --- | --- |
| **Extent of dorsal scale keeling** | Smooth | Strongly keeled on posterior 2/3 of body | Smooth or faintly keeled above vent | Smooth | Smooth | Smooth | Strongly keeled on posterior 1/2–2/3 of body | Smooth | Smooth | Smooth | Keeled above vent | Smooth or keeled on posterior 1/4 of body | Strongly keeled posteriorly |
| **Supraoculars** | Absent | Present | Present | Absent | Present | Present | Present | Present | Present | Absent | Present | Present | Present |
| **Postoculars** | Absent | Present | Present | Absent | Present | Present | Present | Present | Present | Present | Present | Present | Present |
| **1st pair of infralabials** | Broadly separated | In contact | In contact or narrowly separated | Broadly separated | Separated | In contact | In contact | Separated | In contact or narrowly separated | In contact | Broadly separated | In contact or separated | In contact |
| **Ventrals (♂)** | 122–126 | 116–123 | 131–149 | 126–133 | 135–137 | 129 | 114 | 125–130 | 122–138 | 131–138 | 126–132 | 125–139 | – |
| **Ventrals (♀)** | 123–132 | 125–136 | 137–151 | 134–138 | 145–147 | 130–134 | 118–124 | 130 | 128–138 | 136–147 | – | 129–140 | 132 |
| **Subcaudals (♂)** | 35–39 | 45–49 | 36–50 | 40–43 | 34–36 | 32 | 55 | 33–35 | 22–31 | 39–46 | 39+–43+ | 34–39 | – |
| **Subcaudals (♀)** | 29–35 | 37–43 | 31–41 | 32–36 | 24–35 | 27–29 | 49 | 25 | 24–32 | 29–35 | – | 26–31 | 35 |
| **Maxillary teeth** | 09–11 | 10–13 | 10–12 | 10–12 | 10 | 12 | 11–13 | 7 | 11 | 14–17 | 11 | 9 | – |
| **Tail length / total length ratio (♂)** | 0,21 | 0.23–0.24 | 0.17–0.21 | 0.2–0.21 | 0.16–0.18 | 0,17 | 0,24 | 0.15–0.18 | 0.12–0.16 | 0,22 | 0.20+–0.21+ | 0.15–0.18 | – |
| **Dorsal coloration pattern** | Slate black | Dark brownish or grayish black | Brownish or slate black | Dark saddles on a red or white background | Red orange middorsal spots on a dark grayish brown background, darker posteriorly | Brownish | Dark brown | Dark crossbands on a red-orange background | Gray background, ranging from patternless to extensively marked with bands, laterally offset partial bands, and lateral blotches that range from pale grayish cream to brick red | Grayish to reddish brown | Gray (?) | Brownish, one juvenile with a pink collar | Dark brown |
| **Geographic distribution** | Sierra Mixe, eastern Oaxaca, Mexico | Northern Chiapas and southern Veracruz, Mexico to western Guatemala | Central Oaxaca, Mexico | Sierra de Juárez, northern Oaxaca, Mexico | Northwestern El Salvador to southwestern Honduras | Southeastern Chiapas, Mexico and southwestern Guatemala | Sierra de Juárez and Sierra Mixe, northern Oaxaca, Mexico | Sierra de Zongolica, west-central Veracruz, and Sierra de Quimixtlán, east-central Puebla, Mexico | Sierra de Omoa, northwestern Honduras | Eastern Chiapas, Mexico through southern Guatemala to southwestern Honduras and northwestern El Salvador | Sierra Madre del Sur, southern Oaxaca, Mexico | Sierra Madre Oriental, east-central Hidalgo and northern Puebla, Mexico | Isthmus of Tehuantepec, Oaxaca, Mexico |
| **Habitat** | Cloud forest | Cloud forest, pine-oak ofrest, and wet forest | Cloud forest and pine-oak forest | Cloud forest | Cloud forest | Cloud forest and wet forest | Wet forest and cloud forest ecotone | Cloud forest | Cloud forest | Humid pine-oak forest | Humid pine-oak forest | Cloud forest and pine forest | Rain Forest |
